# Effects of Thapsigargin on the Proliferation and Survival of Human Rheumatoid Arthritis Synovial Cells

**DOI:** 10.1155/2014/605416

**Published:** 2014-02-09

**Authors:** Hui Wang, Xiu-zhi Jia, Chun-jie Sui, Yan-ping Zhao, Yi-fang Mei, Yi-ning Zheng, Zhi-yi Zhang

**Affiliations:** ^1^Department of Rheumatology, The First Affiliated Hospital of Harbin Medical University, No. 23, Road Youzheng, Nangang District, Harbin, Heilongjiang 150001, China; ^2^Department of Immunology, Harbin Medical University, No. 157, Road Baojian, Nangang District, Harbin, Heilongjiang 150001, China; ^3^Department of Gerontology, The First Affiliated Hospital of Harbin Medical University, No. 23, Road Youzheng, Harbin, Heilongjiang 150001, China

## Abstract

A series of experiments have been carried out to investigate the effects of different concentrations of thapsigargin (0, 0.001, 0.1, and 1 **μ**M) on the proliferation and survival of human rheumatoid arthritis synovial cells (MH7A). The results showed that thapsigargin can block the cell proliferation in human rheumatoid arthritis synovial cells in a time- and dose-dependent manner. Results of Hoechst staining suggested that thapsigargin may induce cell apoptosis in MH7A cells in a time- and dose-dependent manner, and the percentages of cell death reached 44.6% at thapsigargin concentration of 1 **μ**M treated for 4 days compared to the control. The protein and mRNA levels of cyclin D1 decreased gradually with the increasing of thapsigargin concentration and treatment times. Moreover, the protein levels of mTORC1 downstream indicators pS6K and p4EBP-1 were reduced by thapsigargin treatment at different concentrations and times, which should be responsible for the reduced cyclin D1 expressions. Our results revealed that thapsigargin may effectively impair the cell proliferation and survival of MH7A cells. The present findings will help to understand the molecular mechanism of fibroblast-like synoviocytes proliferations and suggest that thapsigargin is of potential for the clinical treatment of rheumatoid arthritis.

## 1. Introduction

Rheumatoid arthritis (RA) is a systemic autoimmune disease characterized by chronic inflammation of multiple joints, with disruption of joint cartilage. When the disease is unchecked, it leads to substantial disability and premature death. It affects approximately 0.8 percent of adults worldwide, is more common in women (by a ratio of 3 to 1), and has an earlier onset in women, frequently beginning in the childbearing years [1, 2]. An important characteristic of the rheumatoid synovium is the marked hyperplasia of the lining layer, which is caused by an increased number of fibroblast-like synoviocytes (FLSs) and macrophages. In RA synovium, FLSs, the major cell population in invasive pannus, actively participate in the inflammatory processes of RA [3, 4]. RA FLSs proliferate abnormally, show resistance to Fas-mediated apoptosis, and are able to induce cartilage destruction in the absence of other immune cells when adoptively transferred into SCID mice [5, 6]. In contrast, angiogenesis, the process of new blood vessel formation, is also highly active in RA, particularly during the onset of the disease. Furthermore, newly formed vessels can transport inflammatory cells to synovitis sites and supply nutrients and oxygen to the pannus and thus maintain a chronic inflammatory state [7, 8].

D-cyclins are thought to drive cell cycle progression by associating with their catalytic partners (termed cyclin-dependent kinases CDK4 and CDK6) and by guiding these kinases to their cellular substrates [9, 10]. Some findings are consistent with a model in which cyclin D1 serves as a key sensor and integrator of extracellular signals of cells in early to mid-G1 phase, mediating its function through binding both the CDKs and histone acetylase (p300/cAMP response element-binding protein-binding protein (CBP) and P/CAF) and histone deacetylases to modulate local chromatin structure of the genes that are involved in regulation of cell proliferation and differentiation [11, 12]. More recent work indicates that the induction of cyclin D-CDK complexes results in the redistribution of CDK inhibitor p27Kip1 from cyclin E-CDK2 complexes to cyclin D-CDK4y6 complexes, thereby triggering the kinase activity of cyclin E-CDK2 holoenzyme [13]. Thus, D-cyclins also control cell cycle progression in a kinase-independent manner, *via* their interaction with p27Kip1.

The invasive properties of FLS correlate with radiographic and histological damage in RA and pristane-induced arthritis (PIA). Earlier reports showed that highly invasive FLSs obtained from PIA-susceptible DA (blood type D, Agouti) rats have increased expression of genes associated with invasive cancers, including Villin-2/ezrin. Villin-2/ezrin mediates invasion *via* mammalian target of rapamycin (mTOR) [14]. Moreover, the mTOR inhibitor rapamycin to assess the role of the ezrin-mTOR pathway on the invasive properties of FLS has been investigated. Results indicated that rapamycin may prevent actin reorganization in both DA and RA FLS and inhibited the directional formation of lamellipodia. Phosphorylation of the lamellipodia marker FAK was also reduced by rapamycin. Rapamycin significantly reduced RA and DA rat FLS invasion *via *the suppression of the mTOR signaling pathway [15, 16]. These findings suggested that rapamycin may have a role in RA therapy aimed at reducing the articular damage and erosive changes mediated by FLS.

Although it has been proposed that endoplasmic reticulum (ER) stress is involved in the proliferation and invasive properties of FLS [3, 17], the detailed mechanism of ER stress in rheumatoid arthritis still needs to be determined. In the present study, we found that thapsigargin, a well-known inhibitor of ER calcium-ATPase, may inhibit the cell proliferation of MH7A human rheumatoid arthritis synovial cells in a time- and dose-dependent manner. By thapsigargin treatment, the inhibition of cell proliferation was accompanied by the increased cell apoptosis. All these effects of thapsigargin on MH7A cells were mediated by the reduction of cyclin D1 mRNA and protein levels, which were mediated by mTOR. Our findings highlight important roles of cyclin D1 in the proliferation and apoptosis in rheumatoid arthritis and set a stage to the clinical treatment of rheumatoid arthritis by thapsigargin.

## 2. Materials and Methods

### 2.1. Chemical and Materials

The inhibitor of ER calcium-ATPase thapsigargin was purchased from Sigma-Aldric (St. Louis, MO, USA). Dulbecco's Modified Essential Medium (DMEM) and Fetal Bovine Serum (FBS) were purchased from Gibco Invitrogen Corporation (Carlsbad, CA, USA). The Hoechst kit was from Beyotime Biotechnology Co. (Haimen, Jiangsu, China). The cyclin D1 antibody was from Santa Cruze Biotechnology (Santa Cruze, CA, USA). The pS6K and p4EBP1 antibodies were from Cell Signaling Technology (Danvers, MA, USA). And antibeta actin was from Millipore (Billerica, MA, USA). Other chemicals were of the highest purity available.

### 2.2. Assays of Cell Culture

Human MH7A synovial cell is an immortalized cell line by stably transfecting rheumatoid fibroblast-like synoviocyte cells with SV40 T antigen gene. They have already reached over 150 population doublings through culture crisis and have been growing rapidly. MH7A human rheumatoid arthritis synovial cell was chosen as *in vitro* experiment system, which was obtained from Shanghai Institute of Cell Biology (introduced from American Type Culture Collection). In our experiments, MH7A cells were plated in 6-well plates at 1.0 × 10^6^ cells/mL. The cells were incubated in Dulbecco's Modified Essential Medium (DMEM) containing 10% Fetal Bovine Serum (FBS) plus antibiotics for growth in 5% CO_2_ at 37°C.

### 2.3. Pharmacological Manipulations

For pharmacological treatment in MH7A cells, the final concentrations (0.001, 0.1, and 1 *μ*M) of thapsigargin were applied to these cells and then incubated for 2 and 4 days. No additives were used as internal controls. After culturing, the cells were harvested for subsequent sulforhodamine B (SRB) colorimetric assay and Hoechst staining.

### 2.4. RNA Extraction and Real-Time PCR Assay

Total RNA was extracted from MH7A cells using Trizol reagent (Invitrogen, Carlsbad, CA, USA). RNA quality was determined using an Agilent Bioanalyzer 2100. First-strand cDNA was synthesized by random priming using the Quantitect Reverse Transcription kit (Qiagen). Quantitative PCR was performed using a Light Cycler (Roche) with QuantiTect SYBR Green PCR system (Qiagen) and primers for human cyclin D1 (F: 5′-AGCTCCTGTGCTGCGAAGTGGAAAC-3′, R: 5′-AGTGTTCAATGAAATCGTGCGGGGT-3′).

### 2.5. Assay of Ell Proliferation

Cell proliferation was measured using the sulforhodamine B (SRB) colorimetric assay [18]. Briefly, cells were seeded at 1 × 10^3^ cells/well in 96-well microtiter plate with thapsigargin treatment. At various times, cells were fixed in 10% trichloroacetic acid for 1 h at 4°C, rinsed, and subsequently stained for 30 min at room temperature with 0.2% SRB dissolved in 1% acetic acid, followed by air drying. Bound dye was solubilized in 100 *μ*L of 10 mM unbuffered Tris base for 30 min and the OD was read at 490 nm in an ELISA plate reader.

### 2.6. Assay of Hoechst Staining

For the preparation of Hoechst staining, cells were plated with 1.0 × 10^5^ cells/mL in 6-well plates. After pharmacological manipulations, cells were directly stained with Hoechst kit from Beyotime. The cell counting was carried out through the use of the National Institutes of Health software ImageJ.

### 2.7. Assay of Western Blots

Cultured MH7A cells were sonicated with lysis buffer (PBS with 1% Triton X-100 and protease inhibitors). The cell lysate supernatants were harvested by centrifugation at l0000 rpm for 10 min at 4°C. Protein concentrations of the cell supernatants were evaluated and measured by BCA Protein Assay kit (Thermo Fisher Scientific Inc., Rockford, IL, USA). Equal amount of the proteins from each extract was separated in an SDS-polyacrylamide gel (12.6%) with 5% stacking gel in SDS-Tris-glycine running buffer. The proteins were then transferred electrophoretically using a PVDF membrane by standard procedures. The membranes were then blocked by 5% nonfat dry milk in PBST (PBS with 0.1% Tween 20, pH 7.6) for 1 h and probed overnight by proper primary antibodies diluted in PBST at 4°C. The membranes were rinsed 3 times with PBST and incubated with proper secondary antibodies diluted in PBST for 1 h at room temperature. Then, the membranes were rinsed another 3 times with PBST at room temperature for 10 minutes, and proteins were detected by Super Signal enhanced chemiluminescence development (ECL) (Thermo Scientific Pierce) reagent and exposed to films (Kodak). The protein level quantification was carried out by ImageJ.

### 2.8. Statistical Analysis

All statistical analyses were performed by Image software. Quantitative data were shown in *x* ^−^ ± *s* using *t*-tests for comparisons. The values 0.05 (*), 0.01 (**), and 0.001 (***) were assumed as the level of significance for the statistic tests carried out.

## 3. Results

### 3.1. Thapsigargin Inhibits MH7A Cell Proliferation

**Figure 1:**
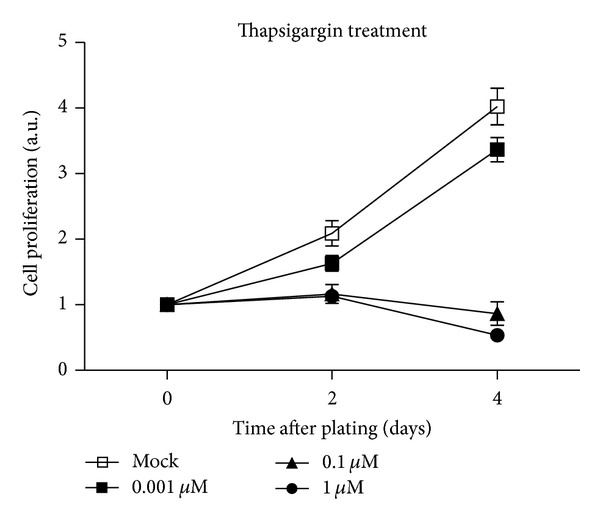
Thapsigargin inhibits MH7A cell proliferation. Histograms showed that the cell proliferation is impaired after thapsigargin treatment (0.001, 0.1, and 1 *μ*M) in MH7A cells, by (SRB) colorimetric assay. Results are averages of three independent experiments.

Thapsigargin has been reported to induce cell death in several types of cells by either increasing the store-mediated calcium entry or ER stress. It has been reported that thapsigargin inhibits replication of human vascular smooth muscle cell at 10 nM concentrations [19]. To determine whether thapsigargin affects cell proliferations in MH7A cells, we quantified cell proliferation in optimal growth conditions over a four-day period using the SRB colorimetric assay. By statistical analysis, we found that thapsigargin exhibited inhibitory effect on cell replications even at 0.001 *μ*M concentration in 2-day and 4-day groups. And higher concentrations of thapsigargin (0.1 *μ*M and 1 *μ*M) showed stronger inhibitory effect on cell replications than those in the control groups (Figure 1). These results suggested that thapsigargin may arrest cell proliferations in MH7A human rheumatoid arthritis synovial cells in a time- and dose-dependent manner.

### 3.2. Thapsigargin Induces Cell Apoptosis in MH7A Cells

**Figure 2:**
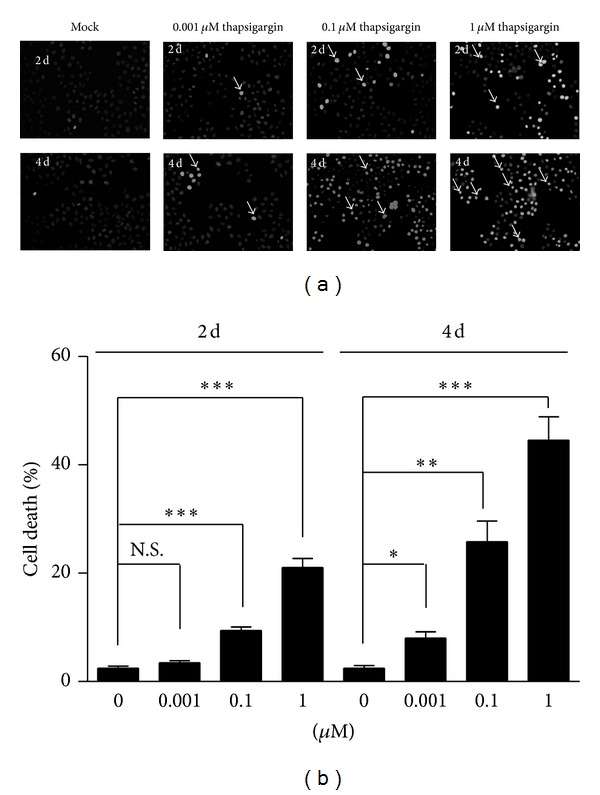
Thapsigargin induces cell apoptosis in MH7A cells. Hoechst stainings (a) and histograms (b) showing the increased cell death (%) after thapsigargin treatment in MH7A cells. Results are averages of three independent experiments. Data represent mean ± SEM. *P < 0.05, **P < 0.01, ***P < 0.001, and N.S.: not statistically significant.

We have shown that thapsigargin may effectively affect cell proliferations in MH7A human rheumatoid arthritis synovial cells. The question is that what is the fate of these nonreplicated cells. Thapsigargin may induce cell apoptosis in several types of cultured cells; we hypothesized that the nonreplicated cells by thapsigargin treatment would be apoptotic. To examine whether thapsigargin induces apoptosis in MH7A human rheumatoid arthritis synovial cells, we applied Hoechst staining to MH7A cells treated by thapsigargin (Figure 2(a)). The results showed that thapsigargin had slight effects on cell death at the final concentration of 0.001 *μ*M (cell death by 3.52%) or 0.1 *μ*M (by 9.43%) for 2 days of treatment (Figure 2(b)). The percentage of cell death increased significantly to 21.1% at the concentration of 1 *μ*M (Figure 2(b)). To examine whether the effects of thapsigargin on cell death of MH7A cells was time-dependent or not, we prolonged the treated time of thapsigargin on MH7A cells to 4 days. Our results suggest that the percentages of cell death increase to 8.06% (0.001 *μ*M), 25.8% (0.1 *μ*M), and 44.6% (1 *μ*M) after thapsigargin treatment (Figure 2(b)). These findings support the notion that thapsigargin would induce cell apoptosis in MH7A cells in a time- and dose-dependent manner.

### 3.3. Thapsigargin Inhibits Cyclin D1 Expression in MH7A Cells

**Figure 3:**
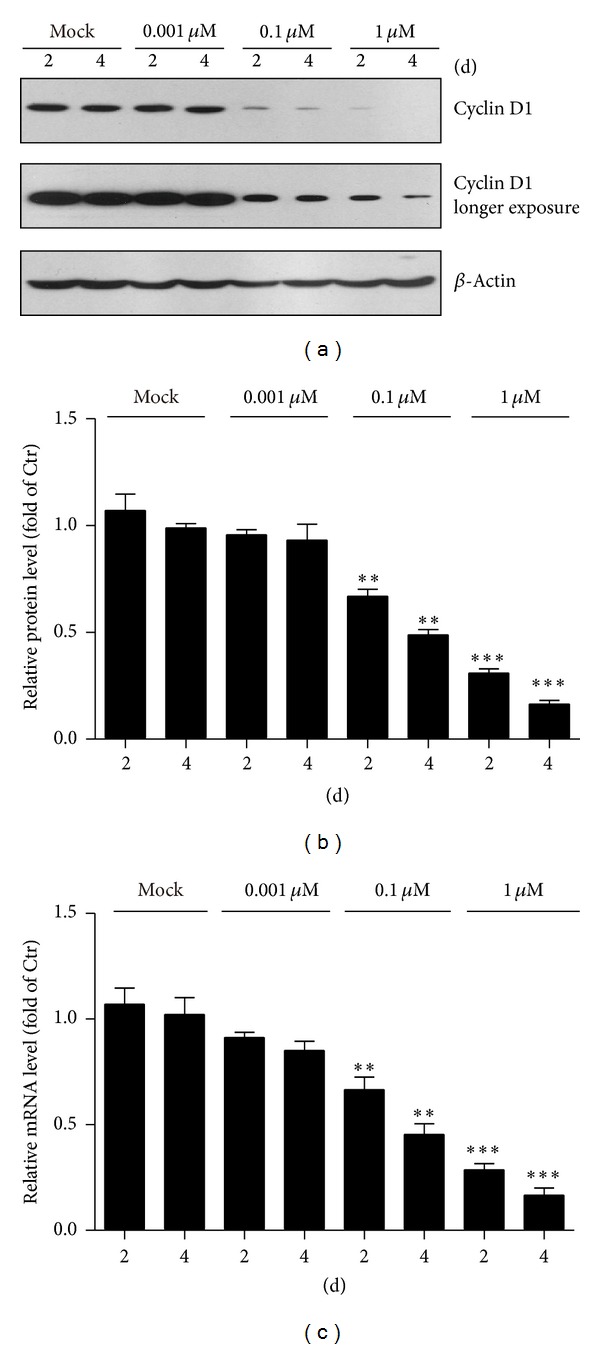
Thapsigargin inhibits cyclin D1 expression in MH7A cells. ((a)-(b)) Western blots and histograms showing that the cyclin D1 protein levels decreased in MH7A cells by thapsigargin treatment. Results are averages of three independent experiments. Data represent mean ± SEM. **P < 0.01 and ***P < 0.001. (c) Real-time PCR results showing that the cyclin D1 mRNA levels decreased in MH7A cells by thapsigargin treatment. Results are averages of three independent experiments. Data represent mean ± SEM. **P < 0.01 and ***P < 0.001.

Earlier reports have shown that thapsigargin may block replication of human vascular smooth muscle cells by suppressing the mRNA and protein expression levels of cyclin D1 [19, 20]. To study the cellular mechanisms of how thapsigargin inhibits cell proliferations and induces cell death in MH7A cells, we focused on the cell cycle factors cyclin D1. Cyclin D1 promotes progression through the G1-S phase of the cell cycle, and loss of cyclin D1 leads to developmental abnormalities, hypoplastic retinas, and pregnancy-insensitive mammary glands in mice [11]. By western blots, we found that cyclin D1 protein levels were reduced to 67.2% and 48.8% after thapsigargin treatment by 0.1 *μ*M for 2 and 4 days and to 30.9% and 16.5% by 1 *μ*M thapsigargin for 2 and 4 days (Figures 3(a) and 3(b)). Moreover, the mRNA levels of cyclin D1 after thapsigargin treatment were analyzed using real-time PCR. The results suggested that the mRNA levels of cyclin D1 were also affected by thapsigargin treatment, and the changes of transcription levels were highly correlated with the quantitative changes of protein levels assayed in MH7A cells (Figure 3(c)). This indicates that thapsigargin may decrease cyclin D1 expressions partly by transcriptional regulation.

### 3.4. Thapsigargin Inhibits Cyclin D1 Expression through mTOR Pathway

**Figure 4:**
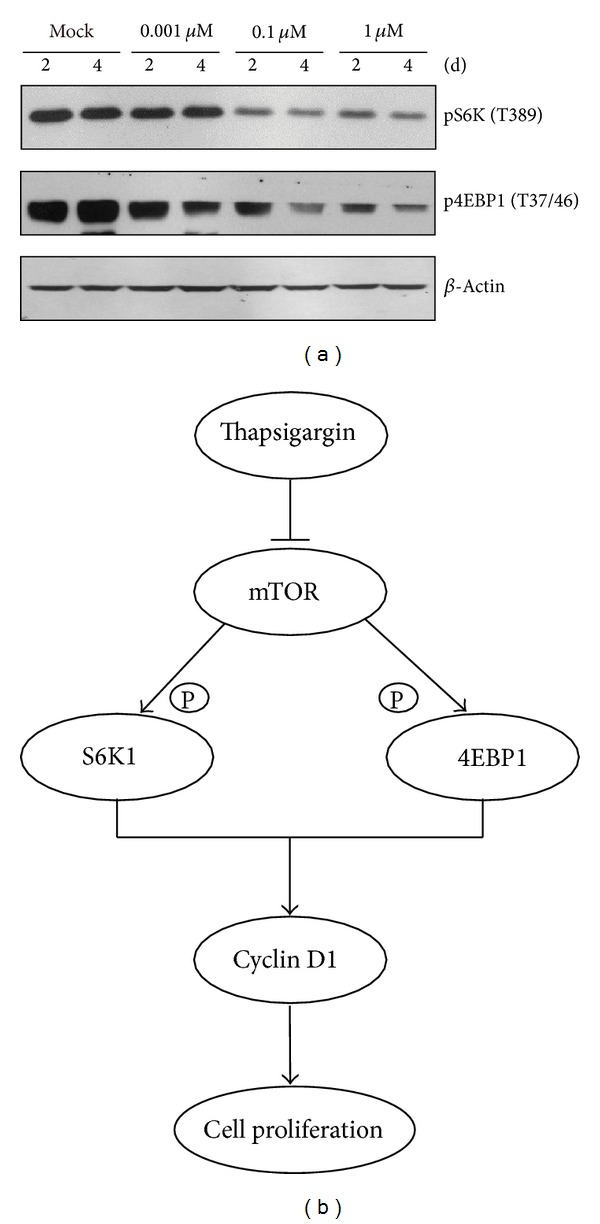
Thapsigargin inhibits cyclin D1 expression through mTOR pathway. (a) Western blots showing that the protein levels mTOR downstream markers pS6K and p4EBP1 were decreased in MH7A cells by thapsigargin treatment. (b) A scheme model highlighting that thapsigargin impairs cyclin D1 expressions by mTOR pathway and finally leads to disrupted cell proliferations and survivals in MH7A human rheumatoid arthritis synovial cells.

To study the molecular mechanism of thapsigargin's effect on cell proliferations and cyclin D1 expressions in MH7A cells, we focused on mTOR pathways. mTOR is a master switch of cell proliferation, growth, and survival and functions in cells at least as two complexes, mTORC1 and mTORC2 [14]. It has been reported that rapamycin, a well-known mTOR inhibitor, inhibited the G1 to S transition by affecting cyclin D1 mRNA and protein stability in NIH 3T3 cells [21]. Thus, to investigate how thapsigargin impairs cyclin D1 expressions, we examined the mTOR activity in thapsigargin treated MH7A cells. Our results revealed that the protein levels of mTORC1 downstream indicators pS6K and p4EBP-1 were both reduced after thapsigargin treatment (0.1 *μ*M and 1 *μ*M) for 2 and 4 days (Figure 4(a)). These results strongly indicate that thapsigargin treatment would impair mTOR activity and lead to cyclin D1 expressions in MH7A cells.

## 4. Discussion

Rheumatoid arthritis, a chronic, systemic, inflammatory autoimmune disease, has as its primary target the synovial tissues. Fibroblast-like synoviocytes (FLSs) have a central role in the formation of the RA pannus and in pannus invasion and destruction of cartilage and bone. Recent advances in understanding the networks that are responsible for the FLS proliferation in rheumatoid arthritis have led to the successful use of therapies [3, 5, 22]. Yet, the regulation of the proliferation properties of FLS remains incompletely understood. Our work clarified that thapsigargin may inhibit the proliferation of MH7A human rheumatoid arthritis synovial cells. Following the addition of thapsigargin, MH7A cells proliferation were inhibited and turned to be apoptotic. Moreover, cyclin D1 mRNA and protein expressions were blocked in the continual presence of thapsigargin. And thapsigargin's effect on cyclin D1 seemed to be mediated by mTOR (Figure 4(b)). These data demonstrate, firstly, that thapsigargin ameliorates cell proliferations in MH7A human rheumatoid arthritis synovial cells, which parallels exactly the cell death. Our work highlights an important role of cyclin D1 in the proliferation and apoptosis in rheumatoid arthritis and sets a stage to the clinical treatment of rheumatoid arthritis by thapsigargin.

Previous studies have interpreted the cytostatic action of 10 nM thapsigargin as being mediated by inhibition of Ca^2+^ ATPase at the ER surface which depletes ER Ca^2+^ pools. And thapsigargin treatment rapidly stops cells from entering the S phase of the cell cycle. Given that thapsigargin can cause sustained elevation in G1 and stop entrance into S phase, it is also reported whether thapsigargin treatment alone may be sufficient to induce apoptosis of androgen-independent prostatic cancer cells without entrance into the S phase [20, 23]. It has been reported that thapsigargin may induce ER stress in rheumatoid and osteoarthritis synovial fibroblasts, and autophagy may prevent cell death induced by thapsigargin [24, 25]. These studies emphasized the role of thapsigargin in the cell proliferations. In the present study, our results suggested that the effects of thapsigargin on the proliferation and survival of human rheumatoid arthritis synovial cells may be distinguished from other reports. These findings firstly revealed the role of thapsigargin on cell death by inhibiting cell proliferation *via* mTOR-cyclin D1 pathway. These results afford us a new thought for the clinical treatment of RA by inhibiting the cell proliferations by thapsigargin.

In untreated cycling cells, cyclin D1 transcript was 9-fold higher than quiescent cells, which is as one would expect. When quiescent cells were stimulated with serum, cyclin D1 mRNA levels increased in a pattern that was paralleled to the elevation in cyclin D1 protein subjected to identical incubation conditions. It has been found that pretreatment of hVSMC with thapsigargin followed by its removal and serum add-back resulted in a delay of 6–15 h in the accumulation of cyclin D1 transcript and cyclin D1 protein. It is concluded, therefore, that the effect of thapsigargin on the overall expression of cyclin D1 is at the promoter level [19, 20]. Now, we reported that thapsigargin dramatically reduced the transcription and translation of cyclin D1 in MH7A human rheumatoid arthritis synovial cells. Because mTOR activity was critical for the cyclin D1 stability, it is reasonable to believe that thapsigargin regulates cyclin D1 expressions by mTOR pathway.

## 5. Conclusion

In summary, the present study suggested that the inhibition of cell proliferation was accompanied by the increased cell apoptosis in MH7A cells by thapsigargin treatment. The reductions of cyclin D1 mRNA and protein levels were also noted after thapsigargin treatment, which was mediated by mTOR. Our findings highlight important roles of cyclin D1 in the proliferation and apoptosis in rheumatoid arthritis and set a stage to the molecular mechanism of FLS proliferations and the clinical treatment of rheumatoid arthritis by thapsigargin.
